# Curcumin Enhances Neurogenesis and Cognition in Aged Rats: Implications for Transcriptional Interactions Related to Growth and Synaptic Plasticity

**DOI:** 10.1371/journal.pone.0031211

**Published:** 2012-02-16

**Authors:** Suzhen Dong, Qingwen Zeng, E. Siobhan Mitchell, Jin Xiu, Yale Duan, Chunxia Li, Jyoti K. Tiwari, Yinghe Hu, Xiaohua Cao, Zheng Zhao

**Affiliations:** 1 Key Laboratory of Brain Functional Genomics, Ministry of Education, Shanghai, Key Laboratory of Brain Functional Genomics, East China Normal University, Shanghai, China; 2 Institutes for Advanced Interdisciplinary Research, East China Normal University, Shanghai, China; 3 Unilever R&D, Vlaardingen, The Netherlands; 4 Unilever R&D, Shanghai, China; 5 Unilever R&D, Bangalore, India; Université Pierre et Marie Curie, France

## Abstract

**Background:**

Curcumin has been demonstrated to have many neuroprotective properties, including improvement of cognition in humans and neurogenesis in animals, yet the mechanism of such effects remains unclear.

**Methodology:**

We assessed behavioural performance and hippocampal cell proliferation in aged rats after 6- and 12-week curcumin-fortified diets. Curcumin enhanced non-spatial and spatial memory, as well as dentate gyrate cell proliferation as compared to control diet rats. We also investigated underlying mechanistic pathways that might link curcumin treatment to increased cognition and neurogenesis via exon array analysis of cortical and hippocampal mRNA transcription. The results revealed a transcriptional network interaction of genes involved in neurotransmission, neuronal development, signal transduction, and metabolism in response to the curcumin treatment.

**Conclusions:**

The results suggest a neurogenesis- and cognition-enhancing potential of prolonged curcumin treatment in aged rats, which may be due to its diverse effects on genes related to growth and plasticity.

## Introduction

The polyphenol compound curcumin is the main component of turmeric curcuminoids derived from tumeric spice, which exhibits many therapeutic properties. Numerous studies have shown that curcumin possesses not only anti-inflammation, anti-oxidative stress, and tumor reduction properties [1], but also neuroprotection against a wide spectrum of neurodegenerative conditions in animal models [2]. Due to its therapeutic potential, curcumin is currently undergoing human clinical trials for treating inflammatory-linked diseases and several types of cancer [1].

In recent years the focus has been shifted to neuroprotective effects of curcumin on cognition. Epidemiologic data has shown that regular curcumin intake may be related to better cognitive function in healthy elderly [3], while in rat models curcumin appears to reverse various forms of cognitive impairment [4], [5], [6], [7], [8], [9]. For example, chronic administration of curcumin can ameliorate age-related spatial memory deficits [10]. These effects may be due to curcumin's activity on oxidative stress [4], [5], [7], BDNF and ERK/P38 kinase signalling pathways [6], degradation of PKCδ [10] or inhibition of histone acetyltransferase [11], as well as several other activities. Curcumin also may protect against Alzheimer's disease (AD) pathology. Both *in vitro* and *in vivo* studies have shown that curcumin prevents amyloid-beta build-up, one of pathological hallmarks of AD [12], [13], [14]. Despite the above observations, understanding of curcumin's diverse neuroprotective activities is still limited, especially how curcumin influences neuronal proliferation.

Adult neurogenesis has been suggested to be an important event for cognitive function [15], [16]. Two recent publications revealed that curcumin enhanced neurogenesis in adult rodents. Xu et al found that oral administration of curcumin increased the proliferation of hippocampal progenitor cells in chronically stressed rats [17], while Kim et al. showed that curcumin could stimulate proliferations of hippocampal neural progenitor cells both at embryonic stage and adult in mice [18]. These studies, however, used relatively young animals (only several weeks old) that have relatively high rates of neurogenesis. Furthermore, neither study investigated behavior, which could have shed light on the functional implications of curcumin-induced neurogenesis. Thus, it is still not clear if neurogenesis is responsible for treatment effects on learning and memory.

The present study investigated the effects of short-term (6-week) and long-term (12-week) curcumin-supplemented diet on hippocampal cellular proliferation and cognitive function in aged rats. A battery of behavioral tests was used to examine the cognitive and psychomotor benefits of such treatment. Hippocampal and cortical transcriptional responses to dietary treatment were assessed via exon array in order to delve further into possible mechanisms of curcumin activity.

## Results

### Effects of curcumin on non-spatial and spatial memory in aged rats

**Figure 1:**
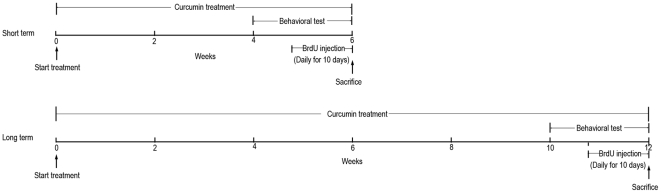
Experimental design of this study. Curcumin was given to the aged rats in food for 6 (short-term) or 12 weeks (long-term). Behavioural tests were performed on the last two weeks of curcumin administration and BrdU were injected daily for ten days on the last ten days. Rats were killed for immunihistological and biochemical analysis at the end of curcumin treatment.

To assess the effects of curcumin on cognitive function, behavioral performance was detected during the last two weeks of 6-week or 12-week curcumin administration (Figure 1). The results from the open field and rota rod tests showed that neither 6- nor 12-week curcumin treatments gave rise to any significant differences between the two groups in locomotor activity, anxiety and motor coordination (Figure S1 and S2).

**Figure 2:**
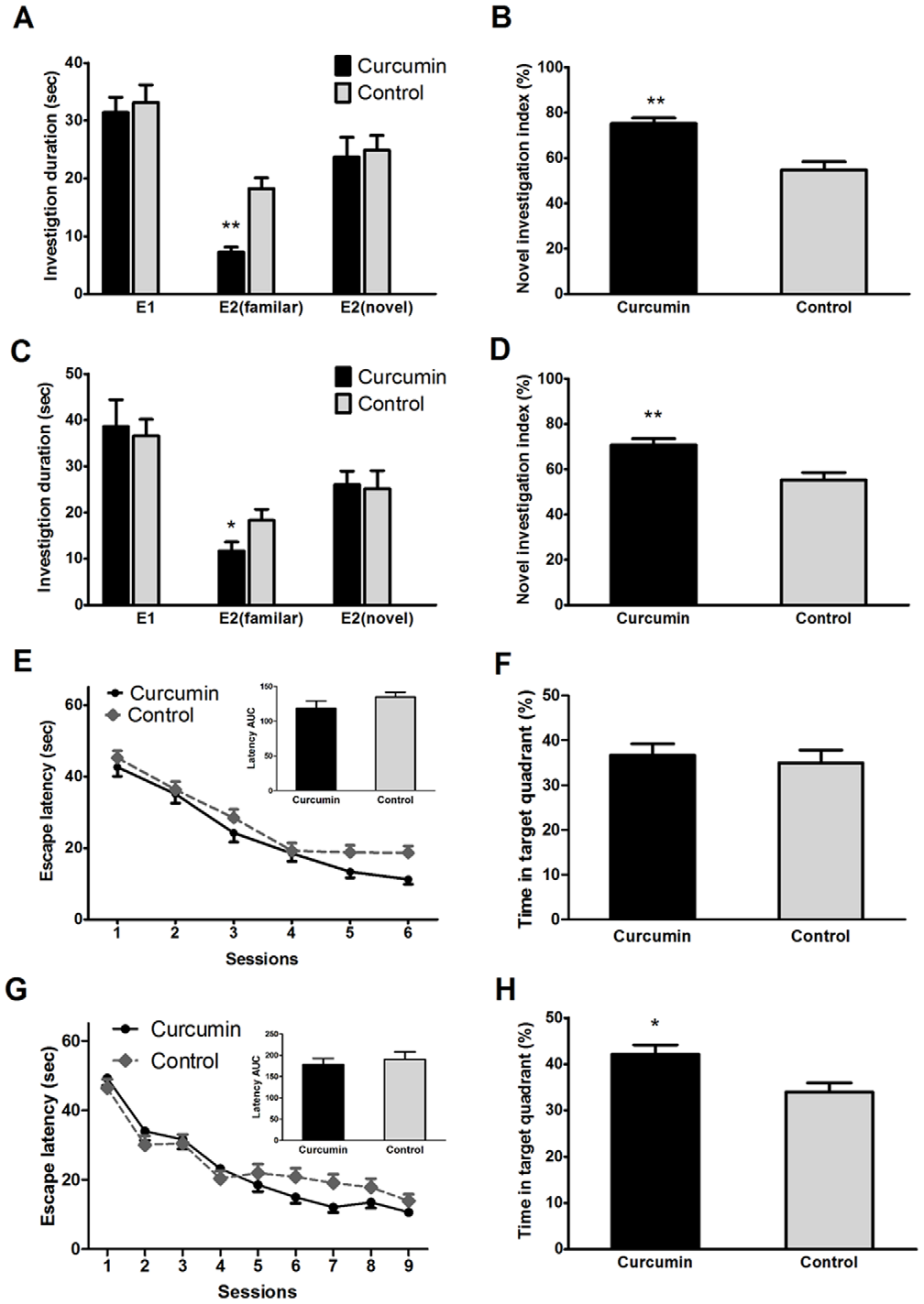
Social recognition ability and spatial reference memory were enhanced after 6- or 12-week curcumin treatment. A and C: In the first trial (Exposure, E1) of social recognition task, the curcumin group and control group had the same preference for the juvenile rats. In the second trial (Exposure 2, E2) of social recognition task, the time spent on exploring the novel and the familiar juvenile rats was expressed as E2_(novel)_ and E2_(familiar)_, respectively. 6- or 12-week curcumin treatment exhibited the significant lower exploration for the familiar juvenile rat during the second trial (E2). B and D: There was significance difference between curcumin and control groups in investigation index after 6- or 12-week treatment with curcumin treatment in aged rats (P<0.01). Investigation index = E2_(novel)_/(E2_(familiar)_+E2_(novel)_). E and G: Effects of 6- and 12-week curcumin treatment during Morris water maze training sessions. Latency to reach the platform and area under curve (AUC) of latencies are shown. Data are expressed as means (± SEM) of daily averages of 4 trials. The area under curve for latency was no significant difference between drug treatment and control for both 6- (Figure E) and 12-week (Figure G, p>0.05). F and H: Effects of 6- and 12-week curcumin treatment during the Morris water maze probe trial. Time spent in the target quadrants is shown. Curcumin group spent more time in the target quadrant than control group after 12-week drug treatment. *P<0.05.

For 6-week treatment, there was a significantly higher social recognition index in curcumin-treated group than that in the control group (Figure 2B). This was demonstrated by the reduced exploration to the familiar juvenile rat (Figure 2A) during the second session (E2, exposure 2) (p<0.01), and the unchanged exploration time during the first session (E1, exposure 1) (Figure 2A). Similarly, the rats from 12-week curcumin-treated group also exhibited a significantly higher novel recognition index than that of the control (p<0.01, Figure 2D). This was mainly due to the lower exploration of the familiar juvenile rat in the curcumin group during the E2 session (Figure 2C). Thus both 6- and 12-week curcumin treatments could significantly improve the social recognition memory of the aged rats.

To determine the effects of curcumin on spatial learning and memory abilities, rats with curcumin treatment were tested on Morris water maze task. Figure 2E and 2G present both the acquisition curves (mean latencies ± SEM to locate the hidden platform) and the area under curve (AUC, Figure 2E and 2G inset) for latencies. There was no significant difference in the area under curve for latencies between the control and the rats with 6- or 12-week curcumin treatment (p>0.05). During the probe test,no significant difference in the amount of swimming time in the target quadrant was observed between the control and the 6-week curcumin-treated groups (p>0.05) (Figure 2F). By contrast, the rats with 12-week curcumin treatment spent a significantly longer time in the target quadrant than that of the control (p<0.05) (Figure 2H). Taken together, these results revealed that the 12-week treatment of curcumin could significantly improve the spatial memory in the aged rats.

### Effects of curcumin on cell proliferation in the dentate gyrus of aged rats

**Figure 3:**
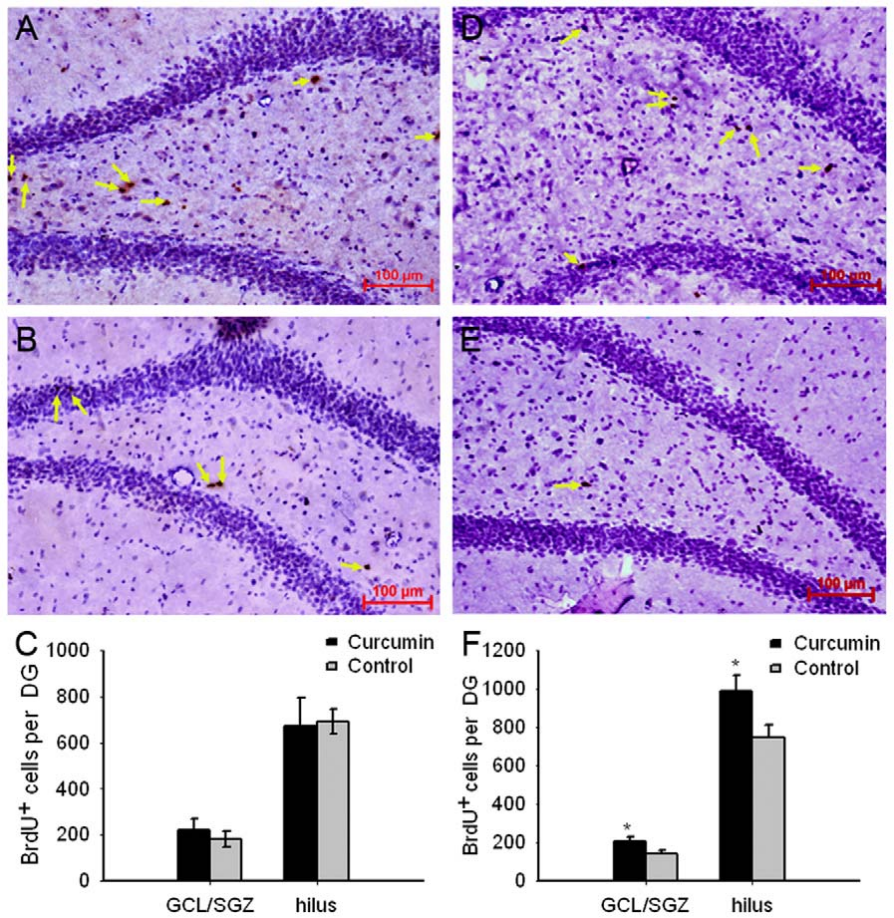
12-week but not 6-week curcumin treatment induced a significant increase of BrdU-positive cells in dentate gyrus. A and B: BrdU immunhistology results from the representative slices of 6-week curcumin-treated rats and controls, respectively. C: the statistical results of 6-week curcumin treatment on hippocampal neurogenesis. D and E: BrdU immunhistology results from the representative slices of 12-week curcumin-treated rats and controls, respectively. F: the statistical results of 12-week curcumin treatment on hippocampal neurogenesis. The yellow arrows: the BrdU-positive cells. In C and F, data were expressed as mean ± SEM. *: P<0.05.

Curcumin-induced hippocampal neurogenesis in the aged rats was examined. Interestingly, BrdU immunostained cells in dentate gyrus were mostly sub-localized in the hilus, rather than the GCL/SGZ (Figure 3A, B, D and E). Quantitative assessment revealed no significant difference in the number of BrdU-labeled cells between 6-week curcumin-treated rats and the control (Figure 3A–C). By contrast, more BrdU-positive cells were observed in both GCL/SGZ and hilus from 12-week curcumin-treated rats as compared to that of the control (Figure 3D–F), suggesting that curcumin treatment for 12-week, but not 6-week, could promote cell proliferation in the dentate gyrus of aged rats.

### Effects of curcumin on gene expression changes in the cortex and hippocampus of aged rats

**Figure 4:**
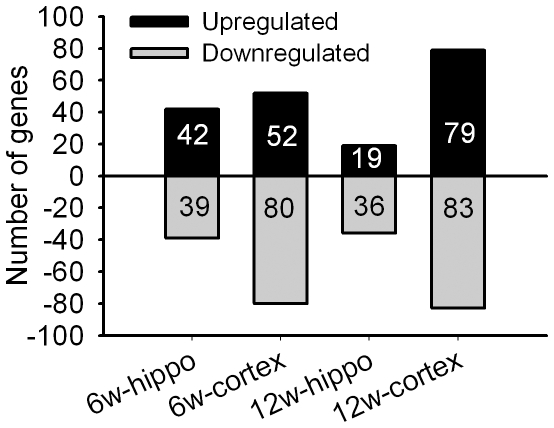
Numbers of up-regulated and down-regulated genes in the hippocampus and cortex after curcumin treatment. Number on the bar showed the number of genes whose expressions were up-regulated or down-regulated. 6 W: 6-week; 12 W:12-week.

**Figure 5:**
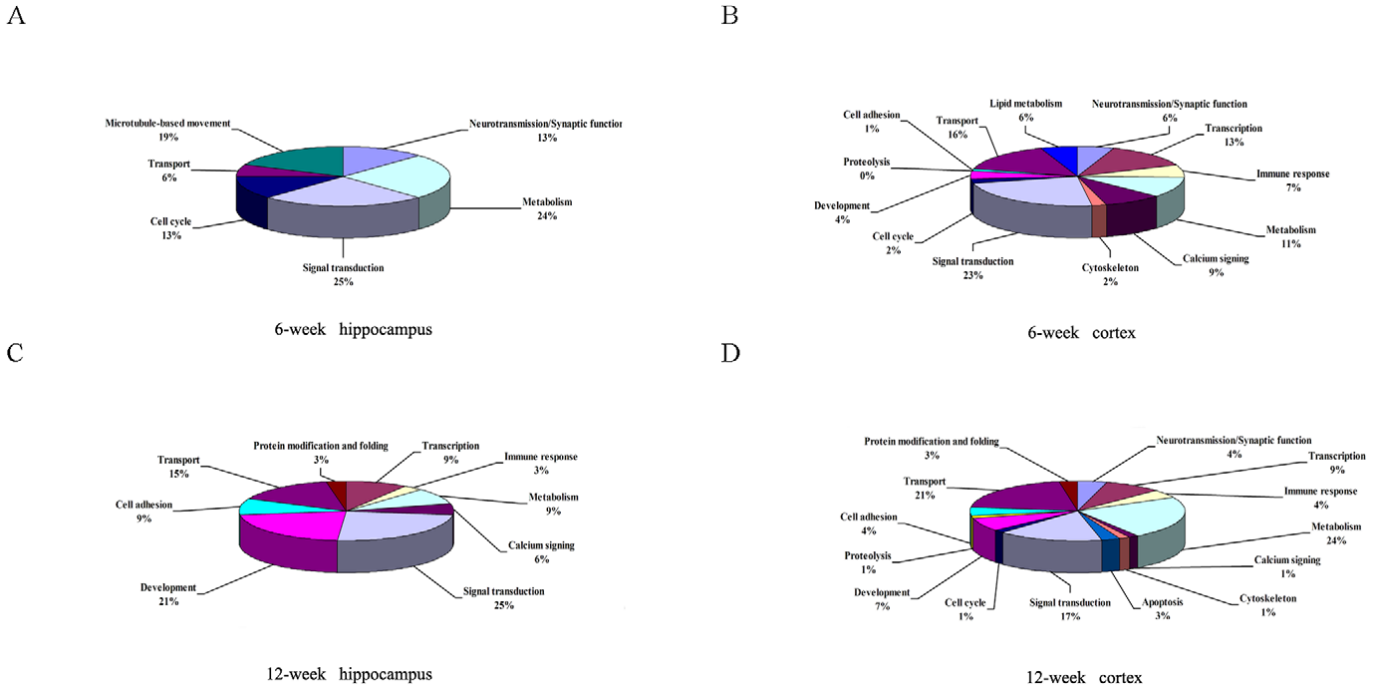
Functional categories of genes affected by curcumin. Percentages of genes whose expression levels are changed by curcumin were indicated.

To further investigate the curcumin-induced changes in the brain and their relationship to the improved cognition observed in the aged rats, we compared the gene expression profiles in the hippocampus and cortex of 6- and 12-week curcumin-treated rats with their corresponding controls. The results showed that the expression of 81 hippocampal genes and 132 cortical genes in 6-week curcumin-treated rats (Figure 4, Table S1 and S2) were consistently and reproducibly changed by 1.5-fold or above, while 55 genes in the hippocampus and 162 genes in the cortex in 12-week curcumin-treated rats (Figure 4, Table S3 and S4) were changed by 1.5-fold. The physiological function analysis of these genes revealed multiple signal pathways in the hippocampus and cortex responsible for curcumin stimulation in modulation of neurotransmission and metabolic homeostasis, neuronal development, signal transduction, transport as well as RNA transcription, and so on (Figure 5). Some functional important genes are summarized in Table 1. The overall gene expression profiles as well as the expressions of individual genes for the cortex and hippocampus were drastically distinct between 6- and 12-week curcumin-treated animals (Table S1, S2, S3, S4). Of them, only two genes (Table 2) showed the same up- or down-regulation in the cortex for the two curcumin treatment periods. Interestingly, there were three genes that showed similar changes in expression level (either increase or decrease) only in one treatment duration (6- or 12-week) and one tissue type (hippocampus or cortex). In addition, there were four other genes that changed commonly to both tissue types (hippocampus and cortex) or both treatment durations (6- and 12-week) but showed opposite regulation in their expression levels (Table 2). Collectively, our data suggested that the molecular compositions of gene expressions triggered by curcumin might relate to the tissue types and the treatment duration we used.

**Table 1:** Representive genes that were differentially expressed in the hippocampus and cortex of the aged rats after curcumin treatment.

Tissue	Treatment duration	Functional classification	Gene	Accession number	Fold change
Hippocampus	6-week	Neurotransmission/Synaptic function	Syt9	NM_053324	1.58
Cortex			Stx1a	NM_053788	1.87
			Cplx3	NM_001109295	1.62
		Transcription	Fezf2	NM_001107251	1.85
			Neurod1	NM_019218	2.3
			Neurod6	NM_001109237	1.86
Hippocampus	12-week	Signal transduction	Adcy1	NM_001107239	1.76
			Kit	NM_022264	0.61
			Htr2c	NM_012765	0.52
		Metabolism	Lpl	NM_012598	0.51
		Development	Wnt2	ENSRNOT00000010427	1.90
			Nnat	NM_053601	0.53
Cortex		Signal transduction	Tiam1	ENSRNOT00000046486	1.61
			Unc5d	NM_001107319	1.87
			Shank3	NM_021676	1.59
			Htr2a	NM_017254	0.33
		Neurotransmission/			
Synaptic function	Nlgn2	NM_053992	1.76		
			Cip98	NM_181088	1.55
		Immune response	Cd74	NM_013069	7
		Cell adhesion	Snip	NM_019378	1.64
		Cell cycle	Wee1	NM_001012742	0.63
		Calcium signing	Cav1	NM_031556	0.55

**Table 2:** Comparison of gene expression regulation in the hippocampus and cortex of the aged rats between 6- and 12-week curcumin treatment groups.

Gene name	Accession number	Fold change			
		6-week treatment		12-week treatment	
		hippocampus	cortex	hippocampus	cortex
Ttc6	ENSRNOT00000032695		1.69		1.67
Bm259	ENSRNOT00000033008	0.53	0.44		
—	ENSRNOT00000033720		1.69		1.80
RGD1311744	ENSRNOT00000036054		0.66	0.51	
RT1-Da	NM_001008847		0.50		5.54
Nts	NM_001102381	1.87	0.34		
Cpne7	NM_001108454			0.40	1.68
Slc38a4	NM_130748	1.75		0.55	
Hydin	XM_226468		0.36	0.59	

**Figure 6:**
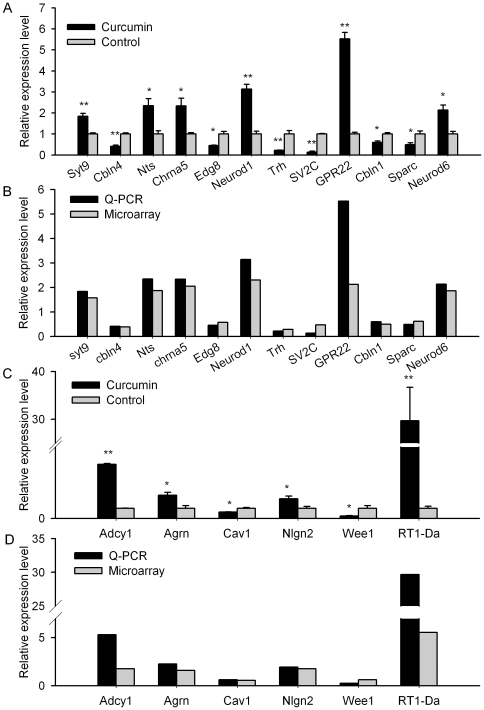
Relative expression levels of relevant genes in the hippocampus of the aged rats. A, B: after 6-week curcumin treatment; C, D: after 12-week curcumin treatment;. A, C: the results of real time PCR; B, D: the comparisons of the relative gene expressions compared to control rats between the results of quantitative PCR and microarray experiments. Student *t*-test, *P<0.05, **P<0.01 compared to control rats.

To corroborate the expression profiling data from the exon array assay, a total of 18 genes from the hippocampus (12 genes from 6-week treatment group and 6 from 12-week group) were selected for quantitative real-time PCR analysis. The results showed that the trends of the changes of all 18 genes were similar to those observed in the microarray data (Figure 6).

## Discussion

The present study demonstrates that prolonged treatment of curcumin enhanced hippocampal neurogenesis in the aged rats. Functional outcomes delivered by curcumin-induced newborn cells may link to a significantly improved cognition, as characterized by a series of behavioural tasks. The transcriptional responses in the cortex and hippocampus also suggest a beneficial role of prolonged treatment of curcumin in modulating neuronal networks that coordinate synaptic plasticity and cell growth.

### Curcumin improved non-spatial and spatial memory in the aged rats

Performance of the rats in behavioural tasks showed that curcumin did not influence the locomotor activity, induce anxiety or improve physical strength in aged rats (Supplementary data). To study cognitive behaviour, we used the social recognition test and Morris water maze test. The social recognition test is a non-spatial learning behavioural paradigm, which primarily employs the olfactory cortex. The discrimination between familiar and novel stimuli is used as an index of learning and memory [19]. Curcumin improved the recognition index after both 6- and 12-week treatment (Figure 2A–D). The results indicated that prolonged curcumin treatment improved the non-spatial memory in the aged rats. Spatial memory refers to the ability of the animal to learn the location of a hidden platform and thus requires hippocampus-based spatial memory [20]. There was a significant improvement in spatial memory after 12-week but not 6-week curcumin treatment (Figure 2E–H). There are a number of studies demonstrating that curcumin improved impaired cognitive function in various disease animal models, including chronically stressed rats [6], [17], diabetic rats [8], cerebral ischemic rats and Mongolian gerbils [21], [22], [23], and AD-like rats [5]. However, the animals in these studies had artificially induced disease conditions and were relatively young (at several months of age). A recently published study showed the effects of curcumin on cognitive improvement in aged healthy Wistar rats [10]. In this study, 18-month-old rats were given 75 mg/kg curcumin by gavage for 8 days, this treatment enhanced polysialated neural cell adhesion molecule (NCAM PSA) expression in the dentate gyrus and significantly improved spatial learning ability and spatial memory [10]. Our results were consistent with this study, but their treatment was much shorter and the curcumin dose was much larger compared to our study. Better spatial memory was only observed after 12-week treatment, suggesting that prolonged curcumin consumption of about 12 mg per day might prevent or slow down the decline of cognitive function with aging.

### Curcumin enhanced cell proliferation of dentate gyrus in the aged rats

It has been widely believed that new neurons arise from the proliferation of neural progenitor cells in the dentate gyrus of hippocampus or subventrical zone in adult animals. Once generated, the cells may undergo processes in terms of morphological and physiological maturation [15], experience-associated regulation [16], [24], [25], [26] and responsiveness to certain stimuli [27], [28], [29], [30]. During the first three weeks of the maturing process, the hippocampal newborn neurons may make specific contributions to brain cognitive functions [16]. In this study, we showed that 12-week, but not the 6-week, curcumin treatment led to enhanced neurogenesis in both the GCL/SGZ and hilus of the hippocampus (Figure 3), suggesting that at the given dosage of curcumin (12 mg per day), the generation of new neuronal cells may require an accumulated effect of the molecule over a prolonged period. Given the relationship between the strengthened memory function and enhanced neurogenesis, our data also suggest that, with specific dosages and treatment durations, curcumin-induced hippocampal cell proliferation may have more predominant effect on spatial memory over non-spatial memory. This is in accord with the specific functions of the hippocampus in mediating spatial memory formation [31]. It is supported also by a previous study, in which a correlation between hippocampal neurogenesis and spatial memory was observed in mice with varied genetic backgrounds [32]. It has often been observed that newborn neurons in the adult brain are initially allocated in the subventricular zone (SVZ) of the lateral ventricle and subgranular zone (SGZ) of the dentate gyrus of hippocampus. In the present study, however, the majority of BrdU-positive cells were found in the hilus, while fewer in the GCL/SGZ, of the dentate gyrus (Figure 3). The reason for the observation is unknown, and more refined studies following the growth, differentiation and migration of these cells and their integration into the circuitry of the adult hippocampus may give hints. Nevertheless, our results suggest a beneficial role of prolonged treatment of curcumin in improving learning and memory, and the underlying mechanism of this role by curcumin may lie on the promotion of neurogenesis in the dentate gyrus, particularily in aged animals. It has to be mentioned that the results from the present study still cannot clarify the functional importance of the curcumin-induced adult neurogenesis. More investigations are needed for exploring the functional developments of the adult hippocampal newborn neurons and their mechanistic relationship with curcumin action towards cognitive improvement.

### Curcumin induced gene expression changes in cortex and hippocampus of the aged rats

Although previous researches have demonstrated important functions of curcumin in cognitive performance [10], possibly via modulations of multiple signaling pathways in the brain [5], [6], [7], [10], [11], [12], [21], [33], there have been no systematic studies on the gene responses to the curcumin treatment in the brain. There have also been no studies on the functional changes of differentially expressed genes stimulated by curcumin in the adult neurogenesis. In this study, we simultaneously examined gene expression changes in the hippocampus and cortex of aged rats after 6- and 12-week curcumin treatment using the arrays containing 27342 rat genes and ESTs. The results showed that the overall profiles of curcumin-triggered gene expression were distinctive. Firstly, the amount of genes that were differentially expressed was larger in the cortex (132 and 162 genes in 6- and 12-week treatment, respectively) than in the hippocampus (81 and 55 genes in 6- and 12-week treatment, respectively) (Figure 4). Secondly, the numbers of up-regulated genes decreased in the hippocampus from the 6- (42 genes) to the 12-week treatments (19 genes) while increased in the cortex from 6- (52 genes) to the 12-week treatments (79 genes) (Figure 4). The influence of the treatment duration on the numbers of down-regulated genes was less in either the hippocampus or the cortex (Figure 4). Thirdly, the individual genes that were differentially expressed and have similar or common functions in the neuronal network as documented were substantially varied according to treatment duration or tissue type (Figure 5, Table S1, S2, S3, S4). Figure 5 show the changes of the representative genes (unknown genes or genes with unknown function are not included in Figure 5). Fourthly, very few differentially expressed genes were influenced both by the treatment duration and tissue type (Table 1). The results thus suggest that curcumin mediates divergent cognition-associated neuronal network activity in a treatment duration- and tissue-dependent manner.

One important finding of the present study, which comes with the functional analysis of the exon array data, is that the majority of those differentially regulated genes have biological and physiological implications in brain development and cognition functions (Figure 5), and some of them are particularly involved in neurogenesis. The NeuroD1 gene, a member of the NeuroD subfamily, is downstream of Wnt signalling pathway and is important for the adult neurogenesis and survival of neuronal progenitors [34]. This gene was over-expressed in the cortex of the 6-week curcumin-treated rats (Table 2). The NeuroD6 gene, the neurogenic bHLH (basic helix-loop-helix) transcription factor, has been previously shown to be involved in regulating neuronal differentiation and energy metabolism [35], and in promoting neuronal survival [36]. The cortical expression of this gene was also found to be up-regulated in this study after 6-week curcumin treatment (Table 2). Similarly, the cortical expression of the Fezf2 gene was increased by about two fold in the rats with 6-week curcumin treatment as compared with the control (Table 2), and the gene has been implicated in patterning as well as neurogenesis [37] and in deciding the fate of subcortical projection neurons [37], [38], [39]. Another group of genes altered by curcurmin, such as Wnt2, Nnat, Tiam1 and Unc5d, have diverse roles in neuron development. The Wnt2 gene has been shown to be involved in dendritic arborization and growth [40], [41] and also the adult neurogenesis [42]. In this study, the expression of this gene was up-regulated in the hippocampus of the aged rats after 12-week curcumin treatment (Table 2). The Tiam1 gene, a Rac1-specific guanine exchange factor, is vital to neurite outgrowth [43] and required for dendritic spine morphogenesis [44]. The 12-week curcumin treatment led to cortical transcriptional activation of this gene (Table 2). These results suggest that the beneficial effects of curcumin in improving cognition of the aged rats might be related to enhancing the adult neurogenesis by the up-regulation of development-associated genes in the brain.

It is interesting to note that several genes that have been shown to participate in neurotransmission and synaptic plasticity were altered in the aged rats after 6- or 12-week curcumin administration. For instance, the hippocampal expression of synaptotagmin IX (Syt9), a synaptic vesicle protein, was increased in the aged rats with 6-week curcumin treatment (Table 2). The functional importance of Syt9 in memory and synaptic plasticity is currently not clear. However, previous studies have demonstrated that other synaptotagmin genes, such as Syt IV and Syt I, are highly associated with associative and spatial memory [45], [46], and long-term potentiation [45], [47]. Meanwhile, we found that expression levels of neurotransmission-associated genes, such as Cplx3 and Stx1a, were also up-regulated in the cortex after 6-week curcumin treatment (Table 2). It has been reported that complexins (Cplxs) facilitate neurotransmitter release at synapses [48], while the syntaxin 1A (Stx1a) gene, which is considered to be essential for synaptic vesicle exocytosis and closely related to synaptic plasticity and consolidation of fear memory [49], was also elevated by curcumin in cortex. There are several genes with documented functions in synaptic transmission and memory formation that were strikingly regulated by 12-week curcumin treatment. These include Adcyl, Kit, and LPL in the hippocampus and Shank3, Cip98, Snip, and Nlgn2 in the cortex (Table 2). Adenylyl cyclase I (Adcy1), which was up-regulated in the hippocampus after the curcumin treatment (Table 2), has been implicated in recognition memory formation [21] and maintenance of remote contextual fear memory [50], and a reduction of its expression level in the hippocampus might greatly contribute to age-related defects in spatial memory [51]. Moreover, converging evidence showed that the administration of Htr2c/Htr2a agonists led to short-term memory and long-term memory impairment [52]. It is interesting to note that 12-week curcumin treatment strongly reduced the expression levels of these two serotonin receptors in the hippocampus (Htr2c) and cortex (Htr2a) of the aged rats, respectively (Table 2). Collectively, these data indicate that the curcumin-induced changes in expression of these neurotransmission-related genes in hippocampus or cortex may greatly contribute to the improvement of cognition in the aged rats.

Alzheimer's disease (AD) is a chronic neurodegenerative disease that is characterized by progressive memory loss. Perhaps the most noticeable feature of the gene profiles in the rats with 12-week curcumin treatment is that the genes that have previously been indicated to participate in AD neurodegenerative processes occupied a major percentage of the total genes altered. For example, previous studies showed that CD74, the invariant chain of class II major histocompatibility complex, might be essential for AD degeneration, and also interacts with amyloid precursor protein (APP) and inhibits beta amyloid production [53]. Interestingly, we observed that expression of CD74 significantly increased by about seven fold in the cortex of rats with 12-week curcumin treatment (Table 2). We also noticed that tyrosine kinase Wee1, which has been reported being a mitotic regulator and can be altered in AD [54], was down-regulated in the cortex of the aged rats after 12-week curcumin treatment (Table 2). Recent studies revealed that dysregulation of cholesterol homeostasis in AD might be due to the increased expression of Cav1 gene leading to alterations of cholesterol distribution in the AD brain [55]. It has also been shown that Cav1 is involved in spatial memory formation and synaptic plasticity [56], [57], and especially plays a role in age-related working memory decline [58]. Consistent with these notions, our data may also suggest a link of the curcumin-induced changes in cortical Cav1 expression to the curcumin-enhanced cognitive function.

In conclusion, the present study demonstrates that curcumin could significantly improve the cognitive function of aged rats. The prolonged treatment of curcumin appears to markedly promote the adult neurogenesis in the dentate gyrus. The unique profiles of the gene expression in both the hippocampus and the cortex induced by 6- and 12-week curcumin treatments were also identified. The biological and physiological function analysis revealed that these genes are important for neuron development/neurogenesis, neurotransmission, synaptic plasticity and memory formation. Taking the above together, our results suggest that prolonged treatment of curcumin might act as a mechanistic mediator governing the adult neurogenesis towards the enhancement of learning and memory. Furthermore, there appeared a transcriptional network interaction in the brain in response to the curcumin treatment, which may also implicate underlying mechanisms of cognitive preservation during aging.

## Materials and Methods

### Ethics statement

All animal experiments described in this study have been conducted according to Animals Act, 2006 (China) and approved by the Institutional Animal Care and Use Committee (IACUC approval ID #M08022) of the East China Normal University

### Animals and treatments

Aged male Sprague-Dawley (SD) rats were purchased from Shanghai SIPPR-BK Laboratory Animal Company, and housed on a 12/12-hr light/dark schedule (lights on at 7:00 AM) in plexiglas cages (two rats per cage) under constant temperature and humidity with *ad libitum* access to rat chow and water. The experimental design is illustrated in Figure 1. Rats were 15-month olds when experimental manipulations began; they were allowed to acclimatize for 2 weeks prior to curcumin treatment. For both short-term and long-term treatments, rats were divided into two groups at random: curcumin and control groups. Curcumin-treated rats received the pelleted curcumin-containing chow ad libitum for 6 weeks (short-term) or 12 weeks (long-term) before they were killed. Curcumin-containing chow was made by mixing curcumin (Sanjivani Phytopharma Pvt. Ltd, India) and standard laboratory chow mix (in powder form), forming a feed with a curcumin concentration of 480 mg/kg. Data from previous studies have shown that rats consume about 25 g chow/day. Thus, the expected daily dosage of curcumin was 12 mg per rat. Control rats received standard laboratory chow during the same period.

### Open field test

Each rat was placed in the center of an opaque plexiglas cage (450×450×400 mm) equipped with the photobeam sensor rings to monitor the locomotor activity of the rat. The rat was allowed to explore the environment for 5 min in the opaque plexiglas cage. Total distance and time travelled by individual at the margin of the cage were measured using a Tru-scan DigBahv-locomotion Activity Analysis System (Coulbourn instrument, USA).

### Rota rod test

The experiment consisted of two phases: a habituation phase and test phase. Habituation was conducted in two days. During the habituation phase, each rat was allowed to stay on the rota rod ( a constantly accelerating rate of 1.5 RPM) for 5 min, facing away from the experimenter. On the test day, rats were individually placed on the rota rod just like the habituation phase. However, each rat had only one chance to finish the task while a constant acceleration was applied to 2 RPM. Latency of each rat to fall from the rota rod was recorded.

### Social recognition test

Rats were brought to the testing room an hour before the first trial. And the housing partner of the rat being tested was removed from the home cage for the duration of the trial. A juvenile rat was introduced to the home cage of the rat being tested and the entire session was videotaped. The length of time spent on sniffing, climbing, grooming or closely following the juvenile was measured during the first trial of three minutes (Exposure 1, E1), after which the juvenile was removed from the home cage of the test rat.

After an inter-trial interval of 30 min, the second trial (Exposure 2, E2) began. The same juvenile from the previous trial and a novel juvenile were introduced to the home cage of the test rat for three min. During the three min, exploration (sniffing, grooming, climbing or closely following) of each juvenile was recorded. The recognition index was calculated as a ratio of the amount of time a rat explored the novel juvenile over the total amount of time the rat explored both juveniles: Investigation index = E2_(novel)_/(E2_(familiar)_+E2_(novel)_).

### Water maze test

The spatial memory function was measured with a hidden-platform water maze. A circular tank 150 cm in diameter and 50 cm in height was filled to a depth of 30 cm with water maintained at 22±1°C, and made opaque by nontoxic black paint. The surface area of the tank was divided into four equal quadrants. An escape platform (15 cm in diameter and 29 cm high) was placed in one of the four quadrants submerged 1 cm below the water surface (25 cm away from the side wall). The tank was surrounded by a black curtain, and was dimly lit. Conditions were kept constant throughout the training and test sessions. In the training session, the rats were gently released into the water, always facing the tank wall. The platform was kept in the same (target) quadrant during the entire training course of the experiment. The rats were trained to find the hidden platform using distal cues available on the curtain (four trials per day). Each trial had a different starting position. Once they found the platform, the rats were permitted to remain on it for 20 s. If the rats did not find the platform within 60 s, they were guided to the platform and also allowed to stay on it for 20 s. Then they were taken out, dried and placed back to the home cage. During each trial session, the time used to reach the hidden platform (escape latency) was recorded. Twenty four hours after the last training day, a probe test was performed to assess memory. During the probe test, the platform was removed from the tank, and the rats were allowed to swim freely. The time rats spent in each quadrant and the swim path were recorded.

### BrdU labelling

Ten days before the end of the treatment period, the curcumin-treated rats and their controls were given BrdU (10 mg/ml freshly prepared in sterile 0.9% NaCl) at a dosage of 75 mg BrdU/kg body weight (one i.p. administration daily for ten days).

### Tissue preparation

Two hour after the final BrdU injection, the rats were deeply anaesthetized with injected pentobarbital (50 mg/kg body weight), and transcardially perfused with 0.9% NaCl solution followed by 4% paraformaldehyde (PFA) in 0.1 M phosphate-buffered saline (PBS). The brains were removed and immediately fixed in 4% PFA-PBS for 2 h, then were immersed in 20% and 30% sucrose-PBS. Subsequently, a total of 80 coronal sections (30 µm in thickness) from each rat were cut by freezing microtome (Leica, Germany). The sections between the anterior landmark where the infrapyrmidal and suprapyramidal blade of the dentate gyrus granule cell layer have formed (about −2.30 mm from Bregama) and the posterior landmark where the dorsal dentate gyrus connects to its ventral part (about −4.80 mm from Bregama) were mounted on microscope slides and stored at −80°C until use.

### Immunohistology

For detection of new-born cells, BrdU immunohistology was performed as previously described [59]. Briefly, slides were incubated with 3% H_2_O_2_ and were rinsed in 0.1 M PBS (pH 7.4) with 0.1% Triton for 5 min. Antigen retrieval was done by keeping the sections in 10 mM sodium citrate buffer (pH 6) at 92–98°C for 20 min. After cooling for 20 min, the sections were rinsed with PBS, and treated with blocking solution (5% goat serum, 3% Triton, 3% BSA in 0.1 M PBS) for 1 h at room temperature and incubated with the BrdU primary antibody (Millipore, 1∶5000 in 0.1 M PBS with 1% BSA) overnight at 4°C. After rinsing consecutively in 0.1 M PBST, sections were incubated with biotinylated goat anti-mouse secondary antibody (1∶150) at 37°C for 30 min. The sections were then thoroughly washed in 0.1 M PBST, and incubated with HRP-labelled streptavidin (1∶150) for 30 min at 37°C. After washing in 0.1 M PBST for 6×5 min, the sections were stained with DAB (1∶20) for 1∼3 min. The slides were rinsed with tap water and counterstained by hematoxylin. The slides were dehydrated, cleared in xylene and mounted with vegetable glue.

### BrdU^+^ cell counting and statistics

The immunostained slices were examined with Nikon microscopy. A cell was thought to be BrdU positive only when it was stained by both hematoxylin and DAB. BrdU-positive cells on every fifth unilateral section through the whole dentate gyrus or subgranular zone (SGZ) were counted at ×400 magnification. Each dentate gyrus was divided into two regions: GCL (granule cell layer)/SGZ and hilus. The cell counting was performed by two different individuals who were blind to the treatment conditions, using the image tool software (Nikon NIS-Elements, USA). The average number of BrdU-labeled cells of the GCL/SGZ or hilus from each slice was multiplied by 80 (there were 80 slices from each rat) to estimate the total number of BrdU-positive cells throughout the dentate gyrus per animal.

### Exon array analysis

The rats (n = 6 per group) were decapitated rapidly and the cortices and hippocampi were dissected. The left and right hippocampus and cortices from each rat were taken apart, and stored at −70°C before use. Half of the hippocampi and cortices from each rat were used for RNA extraction. The hippocampi and cortices of every three rats from one group were pooled together, respectively. Total RNA was extracted using Trizol reagent (Invitrogen, USA) according to the manufacturer's instructions. The RNAs were subsequently purified by QIGEN RNeasy Mini kit. The quantity was determined on a RNA 6000 Nano Labchip using 2100 bioanalyzer (Agilent technologies). Total RNA was used for cDNA synthesis. In vitro transcription was carried out to synthesize cRNA and sense-strand cDNA was synthesized by the reverse transcription using Ambion® WT Expression Kit according to the manufacturer's instructions. Then, single-stranded cDNAs were enzymatically fragmented and labeled using the Affymetrix® GeneChip® WT Terminal Labeling Kit following the manufacturer's instructions. The labeled cDNA was hybridizated to Affymetrix Rat Exon 1.0 ST Array using GeneChip® WT Hybridization, Wash and Stain Kit following the manufacturer's instructions. The hybridization reaction was carried out in hybridization oven 645 (Affymetrix, USA) for 16 h at 45°C at rotation of 60 rpm. After the hybridization and washing, slides were scanned by GeneChip® Scanner 3000 and Command Console Software with default settings. The primary data were generated by Expression Console Software and raw data were normalized by RMA algorithm, Gene Spring Software (Agilent technologies, USA). To identify differential expression genes responding to curcumin treatment, we used fold change method (1.5 fold as a cutoff value). Genes with expression fold changes of ≥1.5 or ≤0.66 were thought to be significantly disturbed genes responsive to curcumin treatment and were extracted for further functional classification analysis. To ensure the reliability of the data, we conducted hybridization experiments in duplicate microarrays from each RNA sample. All data is MIAME compliant and that the raw data has been deposited in GEO database (GSE33137).

### Real time PCR

To validate the microarray results, a series of real time quantitative PCR assays on a group of differentially expressed genes selected from the microarray data were performed. The rationale for choosing these genes was mainly basing on their documented functions in synaptic transmission, learning and memory, and neurogenesis. Total RNA was extracted from the other half of the frozen cortex and hippocampus from each rat using Trizol (Invitrogen, USA). These RNA samples were used to generate cDNA using M-MLV Reverse Transcriptase (Invitrogen, USA). The cDNA samples were used as templates for SYBR Green qPCR using Opticon 2 (MJ research, USA). The primer pairs (Table S5) for the genes were designed using PrimerExpress software and synthesized by Invitrogen (Shanghai, China). Glyceraldehyde-3-phosphate dehydrogenase (GAPDH) was used as endogenous reference. Gene expression level was calculated as 2^−ΔΔCt^ values.

### Statistical analysis

Differences among groups were analyzed using two-tailed *t*-tests or Mann-Whitney U test with the help of the statistical software SPSS. Data were presented as mean ± SEM. Statistical significance was accepted as P<0.05.

## Supporting Information

Figure S1 No effects of curcumin on margin time and margin distance in open field test in the rats with 6-week (A) or 12-week (B) curcumin treatment. Mean ± SEM. There were 15 rats in each group. P.>0.05.
(DOC)

Figure S2 No effects of curcumin on fall time in rota rod test in the rats with 6-week (A) or 12-week (B) curcumin treatment. Mean ± SEM. There are 15 rats in each group. P>0.05.
(DOC)

Table S1 Differentially expressed genes in the hippocampus of the aged rats after 6-week curcumin treatment.
(DOC)

Table S2 Differentially expressed genes in the cortex of the aged rats after 6-week curcumin treatment.
(DOC)

Table S3 Differentially expressed genes in the hippocampus of the aged rats after 12-week curcumin treatment.
(DOC)

Table S4 Differentially expressed genes in the cortex of the aged rats after 12-week curcumin treatment.
(DOC)

Table S5 Primers used in the Real-Time quantitative PCR experiments.
(DOC)
