# Supplementary material for: Curcumin Enhances Neurogenesis and Cognition in Aged Rats: Implications for Transcriptional Interactions Related to Growth and Synaptic Plasticity
Source: PLoS One. 2012 Feb 16;7(2):e31211. doi: 10.1371/journal.pone.0031211 (PMC3281036; doi:10.1371/journal.pone.0031211)
Supplement: Figure S1 — No effects of curcumin on margin time and margin distance in open field test in the rats with 6-week (A) or 12-week (B) curcumin treatment. Mean ± SEM. There were 15 rats in each group. P.>0.05. (DOC) [file pone.0031211.s001.doc]

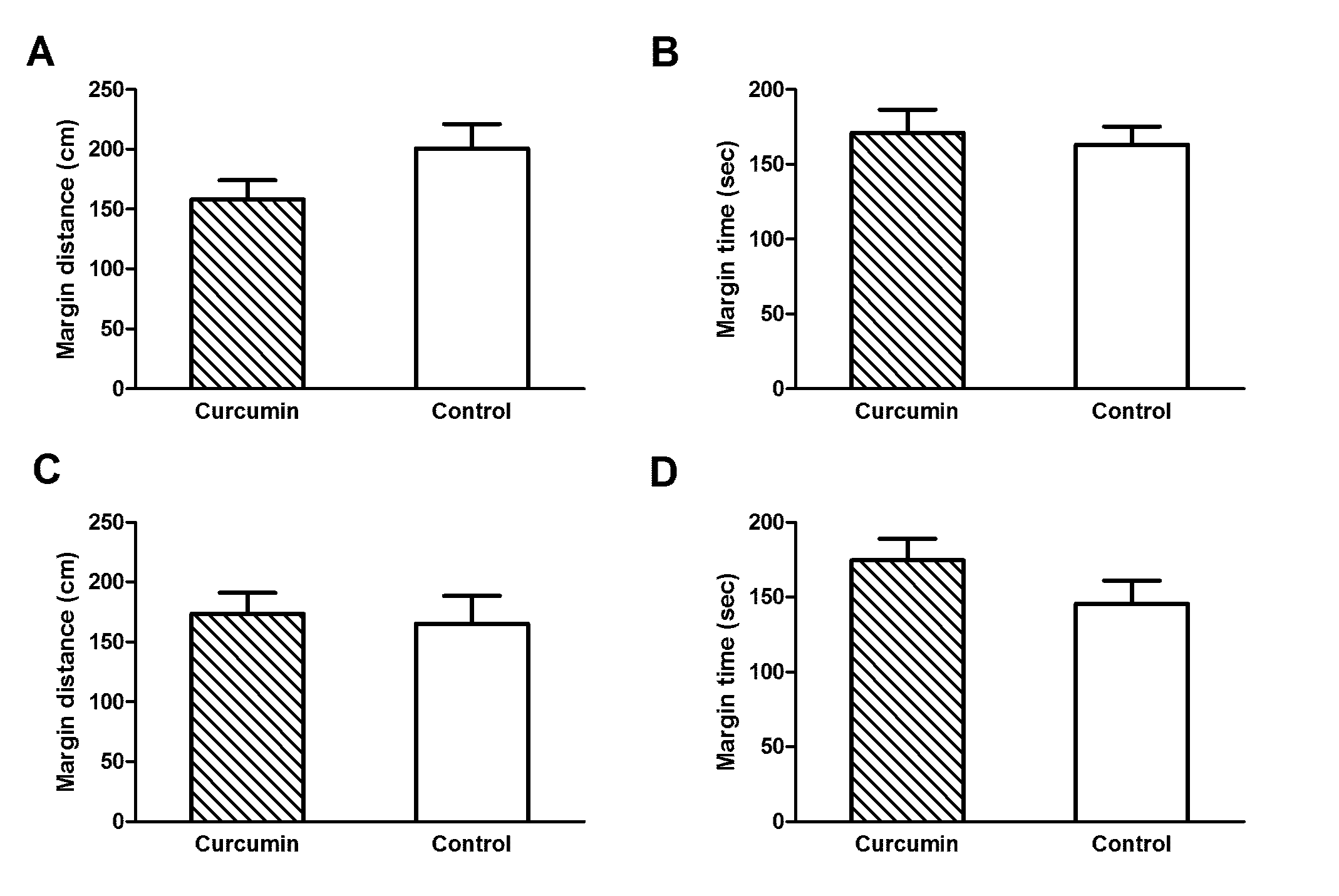


Figure S1. No effects of curcumin on margin time and margin distance in open field test after 6-week (A) or 12-week treatment (B) with curcumin in aged rats. Mean ± SEM, There are 15 rats in each group. ^*^*P* < 0.05.
