# Supplementary material for: Curcumin Enhances Neurogenesis and Cognition in Aged Rats: Implications for Transcriptional Interactions Related to Growth and Synaptic Plasticity
Source: PLoS One. 2012 Feb 16;7(2):e31211. doi: 10.1371/journal.pone.0031211 (PMC3281036; doi:10.1371/journal.pone.0031211)
Supplement: Table S1 — Differentially expressed genes in the hippocampus of the aged rats after 6-week curcumin treatment. (DOC) [file pone.0031211.s003.doc]

Table S1. Differentially expressed genes in the hippocampus of the aged rats after 6-week curcumin treatment.

| **Functional classes** | **Gene name** | **Accession number** | **Fold change** |
| --- | --- | --- | --- |
| Neurotransmission/Synaptic function | Syt9 NM_053324 1.58 | NM_053324 | 1.58 |
|  | Cbln1 | NM_001109127 | 0.50 |
|  | Cbln4 | NM_001109210 | 0.39 |
| Signal transduction | Met | NM_031517 | 1.90 |
|  | Olr1376 | NM_214827 | 0.66 |
|  | Olr1571 | ENSRNOT00000034210 | 0.55 |
|  | Cbln1 | NM_001109127 | 0.50 |
| Metabolism | Nts | NM_001102381 | 1.87 |
|  | Htra4 | NM_001107321 | 0.44 |
|  | Bbox1 | NM_022629 | 0.56 |
|  | Ak7 | NM_001108055 | 0.60 |
| Transport | Slc38a4 | NM_130748 | 1.75 |
| Cytokinesis/cell cycle | Prc1 | NM_001107529 | 0.59 |
|  | Aurkb | NM_053749 | 0.43 |
| Microtubule-based movement | Dnah3 | ENSRNOT00000055066 | 0.47 |
|  | Bm259 | ENSRNOT00000033008 | 0.53 |
|  | Dnahc6 | ENSRNOT00000020894 | 0.58 |
