# Supplementary material for: Curcumin Enhances Neurogenesis and Cognition in Aged Rats: Implications for Transcriptional Interactions Related to Growth and Synaptic Plasticity
Source: PLoS One. 2012 Feb 16;7(2):e31211. doi: 10.1371/journal.pone.0031211 (PMC3281036; doi:10.1371/journal.pone.0031211)
Supplement: Table S2 — Differentially expressed genes in the cortex of the aged rats after 6-week curcumin treatment. (DOC) [file pone.0031211.s004.doc]

Table S2. Differentially expressed genes in the cortex of the aged rats after 6-week curcumin treatment.

| **Functional classes** | **Gene name** | **Accession number** | **Fold change** |
| --- | --- | --- | --- |
| Neurotransmission/Synaptic function | Stx1a | NM_053788 | 1.87 |
|  | Rnf39 | NM_134374 | 1.67 |
|  | Cplx3 | NM_001109295 | 1.62 |
|  | Sv2c | NM_031593 | 0.47 |
|  | Cart | NM_017110 | 0.50 |
| Transcription | Tshz3 | NM_001107506 | 1.62 |
|  | Fezf2 | NM_001107251 | 1.85 |
|  | Pbx3 | NM_001107834 | 0.30 |
|  | Neurod1 | NM_019218 | 2.30 |
|  | Neurod6 | NM_001109237 | 1.86 |
|  | Klf10 | NM_031135 | 1.62 |
|  | Satb2 | NM_001109306 | 1.98 |
|  | Zfp36 | NM_133290 | 0.59 |
|  | Isl1 | NM_017339 | 0.58 |
|  | Meis2 | NM_001107758 | 0.48 |
|  | Dlx5 | NM_012943 | 0.49 |
|  | St18 | NM_153310 | 0.56 |
| Transport | Slc17a7 | NM_053859 | 1.68 |
|  | Kcnt2 | NM_198762 | 1.72 |
|  | Mfsd4 | NM_001109069 | 1.78 |
|  | Cadps2 | XM_001060172 | 1.75 |
|  | Slc16a7 | NM_017302 | 1.58 |
|  | Kcnv1 | NM_021697 | 1.66 |
|  | Chrna5 | NM_017078 | 2.05 |
|  | Scn9a | NM_133289 | 0.58 |
|  | Slc6a9 | NM_053818 | 0.54 |
|  | Rbp1 | NM_012733 | 0.60 |
|  | LOC689064 | NM_001111269 | 0.37 |
|  | Hbb | NM_033234 | 0.42 |
|  | MGC72973 | NM_198776 | 0.36 |
|  | Hba-a2 | NM_013096 | 0.47 |
| Metabolism | Hs3st2 | NM_181370 | 1.70 |
|  | Mpped1 | ENSRNOT00000014720 | 1.73 |
|  | St6gal2 | AJ627626 | 1.93 |
|  | Aspa | NM_024399 | 0.55 |
|  | Adamts4 | AB042272 | 0.58 |
|  | N4bp2 | ENSRNOT00000031792 | 0.62 |
|  | Ptpro | NM_017336 | 0.53 |
|  | Mgst1 | NM_134349 | 0.61 |
|  | Trh | NM_013046 | 0.29 |
|  | Dnahc11 | ENSRNOT00000007233 | 0.57 |
| Inflammation | Vip | BC158798 | 1.65 |
|  | Zfp36 | NM_133290 | 0.59 |
|  | RT1-Da | NM_001008847 | 0.50 |
|  | Scn9a | NM_133289 | 0.58 |
|  | Cd163 | NM_001107887 | 0.45 |
|  | Scg2 | NM_022669 | 0.61 |
| Calcium signaling | Doc2a | NM_022937 | 1.65 |
|  | Nptx1 | NM_153735 | 1.80 |
|  | Stx1a | NM_053788 | 1.87 |
|  | Cadps2 | XM_001060172 | 1.75 |
|  | Calb2 | NM_053988 | 0.40 |
|  | Calcr | NM_053816 | 0.55 |
|  | Pde1c | NM_031078 | 0.54 |
|  | Cacna2d2 | NM_175592 | 0.58 |
| Signal transduction | Gna14 | NM_001013151 | 1.57 |
|  | Rapgefl1 | BC091361 | 1.67 |
|  | Hunk | ENSRNOT00000002866 | 1.59 |
|  | Rasl10a | NM_001108862 | 1.67 |
|  | Npy1r | NM_001113357 | 1.73 |
|  | Arhgap25 | NM_001109247 | 1.59 |
|  | Sstr1 | NM_012719 | 1.67 |
|  | Gpr22 | NM_001106722 | 2.13 |
|  | Chrna5 | NM_017078 | 2.05 |
|  | Sparc | NM_012656 | 0.62 |
|  | Ednrb | NM_017333 | 0.62 |
|  | Stoml3 | NM_001106431 | 0.57 |
|  | P2ry1 | NM_012800 | 0.58 |
|  | Chn2 | NM_032084 | 0.59 |
|  | Olr990 | NM_001006597 | 0.64 |
|  | Cryab | NM_012935 | 0.51 |
|  | Edg8 | NM_021775 | 0.58 |
|  | Igfbp5 | NM_012817 | 0.50 |
|  | RGD1561963 | ENSRNOT00000038528 | 0.60 |
|  | RGD1565967 | ENSRNOT00000009959 | 0.45 |
|  | Gpr101 | NM_001108258 | 0.52 |
| Development | Dact2 | NM_001107464 | 1.59 |
|  | Fezf2 | NM_001107251 | 1.85 |
|  | Neurod1 | NM_019218 | 2.30 |
|  | Neurod6 | NM_001109237 | 1.86 |
| Structure | Argbp2 | NM_053770 | 1.63 |
|  | Mal | NM_012798 | 0.56 |
| Lipid metabolism | Fa2h | ENSRNOT00000025625 | 0.60 |
|  | Ugt8a | NM_019276 | 0.60 |
|  | Fabp7 | NM_030832 | 0.55 |
|  | RGD1312038 | BC127545 | 0.51 |
|  | Nts | NM_001102381 | 0.34 |
| Cell adhesion | Cldn11 | NM_053457 | 0.51 |
| Cell cycle | Fam83d | NM_001107796 | 0.62 |
|  | Anln | ENSRNOT00000024361 | 0.52 |
