# Supplementary material for: Curcumin Enhances Neurogenesis and Cognition in Aged Rats: Implications for Transcriptional Interactions Related to Growth and Synaptic Plasticity
Source: PLoS One. 2012 Feb 16;7(2):e31211. doi: 10.1371/journal.pone.0031211 (PMC3281036; doi:10.1371/journal.pone.0031211)
Supplement: Table S3 — Differentially expressed genes in the hippocampus of the aged rats after 12-week curcumin treatment. (DOC) [file pone.0031211.s005.doc]

Table S3. Differentially expressed genes in the hippocampus of the aged rats after 12-week curcumin treatment.

| **Functional classes** | **Gene name** | **Accession number** | **Fold change** |
| --- | --- | --- | --- |
| Signal transduction | Adcy1 | NM_001107239 | 1.76 |
|  | RGD1561955 | ENSRNOT00000044063 | 1.7 |
|  | Npy2r | NM_023968 | 0.41 |
|  | Odz4 | ENSRNOT00000015181 | 0.63 |
|  | Dgkg | NM_013126 | 0.66 |
|  | Kit | NM_022264 | 0.61 |
|  | Cib2 | NM_001015010 | 0.63 |
|  | Htr2c | NM_012765 | 0.52 |
|  | RGD1565014 | ENSRNOT00000058930 | 1.56 |
| Metabolism | Lpl | NM_012598 | 0.51 |
|  | Car12 | NM_001080756 | 0.62 |
|  | Stxbp6 | ENSRNOT00000005618 | 1.61 |
| Transport | Kcnh5 | NM_133610 | 1.77 |
|  | Kcnj16 | NM_053314 | 0.52 |
|  | Nkain3 | NM_001109540 | 0.56 |
|  | Slc38a4 | NM_130748 | 0.55 |
|  | Igsf4d | NM_001047102 | 1.65 |
| Development | Robo3 | NM_001108135 | 1.63 |
|  | Wnt2 | ENSRNOT00000010427 | 1.90 |
|  | Slit2 | AF141386 | 0.54 |
|  | Hydin | XM_226468 | 0.59 |
|  | Nnat | NM_053601 | 0.53 |
|  | Tgfb3 | NM_013174 | 0.45 |
|  | Stom | ENSRNOT00000025904 | 1.83 |
| Protein homooligomerization | Wfs1 | NM_031823 | 2.19 |
| Calcium ion homeostasis | Ednra | NM_012550 | 0.52 |
| Calcium signaling | Cd3e | NM_001108140 | 2.52 |
| Inflammation | Itgbl1 | NM_001017505 | 0.38 |
| Cell adhesion | Nell1 | NM_031069 | 0.63 |
|  | Lum | NM_031050 | 0.58 |
|  | Plagl1 | NM_012760 | 0.4 |
| Transcription | Nr2f2 | NM_080778 | 0.61 |
|  | Rxrg | NM_031765 | 0.58 |
