# Supplementary material for: Curcumin Enhances Neurogenesis and Cognition in Aged Rats: Implications for Transcriptional Interactions Related to Growth and Synaptic Plasticity
Source: PLoS One. 2012 Feb 16;7(2):e31211. doi: 10.1371/journal.pone.0031211 (PMC3281036; doi:10.1371/journal.pone.0031211)
Supplement: Table S4 — Differentially expressed genes in the cortex of the aged rats after 12-week curcumin treatment. (DOC) [file pone.0031211.s006.doc]

Table S4. Differentially expressed genes in the cortex of the aged rats after 12-week curcumin treatment.

| **Functional classes** | **Gene name** | **Accession number** | **Fold change** |
| --- | --- | --- | --- |
| Neurotransmission/Synaptic function | Nlgn2 | NM_053992 | 1.76 |
|  | Cip98 | NM_181088 | 1.55 |
|  | Agrn | NM_175754 | 1.61 |
| Transcription | Polr2a | ENSRNOT00000046484 | 1.64 |
|  | Sp2 | NM_001107045 | 1.58 |
|  | Nr4a2 | NM_019328 | 2.16 |
|  | Hist1h4m | ENSRNOT00000042564 | 1.57 |
|  | Zfp189 | NM_001107930 | 0.64 |
|  | Bzw1 | NM_198789 | 0.6 |
| Immune response | Cd74 | NM_013069 | 7 |
|  | RT1-Ba | NM_001008831 | 2.31 |
|  | RT1-Db1 | NM_001008884 | 1.75 |
| Cell cycle | Wee1 | NM_001012742 | 0.63 |
| Protein modification | Ttll9 | NM_001014051 | 1.7 |
| Calcium ion homeostasis/ Calcium signing | Cav1 | NM_031556 | 0.55 |
| Transport | Crabp2 | NM_017244 | 1.66 |
|  | Slc28a2 | ENSRNOT00000024658 | 1.55 |
|  | ATP8 | ENSRNOT00000046201 | 2.45 |
|  | Slc35a5 | ENSRNOT00000002853 | 0.61 |
|  | Slc15a2 | NM_031672 | 0.55 |
|  | Mcart1 | NM_001024785 | 0.62 |
|  | Hiatl1 | NM_001107334 | 0.58 |
|  | Vps35 | NM_001105718 | 0.61 |
|  | Slc25a44 | NM_001108947 | 0.57 |
|  | Cav1 | NM_031556 | 0.55 |
|  | RGD1566215 | ENSRNOT00000014734 | 0.61 |
|  | Slc35b4 | NM_001106590 | 0.59 |
|  | Mobkl3 | NM_133528 | 0.58 |
|  | Slc40a1 | NM_133315 | 0.6 |
| Apoptosis | Egln3 | NM_019371 | 0.63 |
|  | Stk17b | NM_133392 | 0.52 |
| Signal transduction | Prkcc | NM_012628 | 1.71 |
|  | Mark4 | ENSRNOT00000023392 | 1.6 |
|  | Tiam1 | ENSRNOT00000046486 | 1.61 |
|  | RGD1562674 | XM_001080090 | 1.64 |
|  | Farp1 | NM_001107287 | 1.75 |
|  | Unc5d | NM_001107319 | 1.87 |
|  | Map3k9 | ENSRNOT00000009693 | 1.71 |
|  | Shank3 | NM_021676 | 1.59 |
|  | Htr2a | NM_017254 | 0.33 |
|  | F2r | NM_012950 | 0.59 |
|  | Cdkl4 | ENSRNOT00000061975 | 0.53 |
| Metabolism | RGD1308168 | XM_001081768 | 1.66 |
|  | Ube2o | ENSRNOT00000030435 | 1.52 |
|  | Wbscr17 | NM_001025112 | 1.66 |
|  | Crabp2 | NM_017244 | 1.66 |
|  | Capns1 | NM_017118 | 0.61 |
|  | Psat1 | NM_198738 | 0.53 |
|  | Insig2 | NM_178091 | 0.65 |
|  | Sc4mol | NM_080886 | 0.63 |
|  | Aadat | NM_017193 | 0.53 |
|  | Enpp6 | NM_001107311 | 0.54 |
|  | Pter | NM_022224 | 0.55 |
|  | Amd1 | NM_031011 | 0.6 |
|  | Rfk | NM_001014106 | 0.63 |
|  | Cml5 | BC158648 | 0.63 |
|  | Azin1 | NM_022585 | 0.63 |
|  | Maoa | ENSRNOT00000003910 | 0.66 |
| Development | Unc5d | NM_001107319 | 1.87 |
|  | Mab21l1 | NM_001109497 | 1.54 |
|  | Tapt1 | ENSRNOT00000058788 | 0.64 |
|  | Amd1 | NM_031011 | 0.6 |
|  | Cml5 | BC158648 | 0.63 |
| Proteolysis | Nrip3 | NM_001108498 | 0.61 |
| Cell adhesion | Snip | NM_019378 | 1.64 |
|  | Pcdh21 | NM_053572 | 0.55 |
|  | Cml5 | BC158648 | 0.63 |
| Cytoskeleton | Abba-1 | ENSRNOT00000023645 | 1.84 |
| Protein modification and folding | Wbscr18 | NM_001109024 | 0.56 |
|  | Adprhl1 | NM_001013054 | 0.56 |
