# Supplementary material for: Curcumin Enhances Neurogenesis and Cognition in Aged Rats: Implications for Transcriptional Interactions Related to Growth and Synaptic Plasticity
Source: PLoS One. 2012 Feb 16;7(2):e31211. doi: 10.1371/journal.pone.0031211 (PMC3281036; doi:10.1371/journal.pone.0031211)
Supplement: Table S5 — Primers used in the Real-Time quantitative PCR experiments. (DOC) [file pone.0031211.s007.doc]

Table S5. Primers used in Real-Time quantitative PCR experiment.

| **Gene name** | **Forward primer** | **Reverse primer** |
| --- | --- | --- |
| Cbln4 | CAAGACTCGCATCATTTACT | GATGGTTTGGCTCTGGTA |
| Gapdh | TATCGGACGCCTGGTTAC | CTGTGCCGTTGAACTTGC |
| Cbln1 | ACTGCTCTGGGCATCTCTGT | AGTGCTGCGTTCTGAGTCAA |
| Chrna5 | CTCTGCTGCCAAACATGAAG | ATTCCTGCTTCAACCAGACG |
| Edg8 | GGAGGGACTCGCCTAGATTC | TCTGTAGCCAGCCACTCCTT |
| GPR22 | CCATAAAGCAACAGCGAACA | GTCCTCAATGTCATCTCGCA |
| Neurod1 | AGGCACGTCAGTTTCACCA | TCCAAAGGCAGTAACGACA |
| Neurod6 | AGGAGACGATGCGACACTC | TCGGGCATTACGACAGACT |
| Sparc | TGGAACATTGCACCACTCG | GAGGCTTGAACTTAGATCACCA |
| SV2C | CCATCTCAGCGTGGAACTC | TGACCAAGGAGCCGAATAT |
| Syt9 | CATGGACTATGACCGTGTAGG | GGGATATGACAGCATCTCACTC |
| Adcyl | GCCTACGCAAGTGGCAGTA | GTTCAAGCACGCAAGGGTT |
| Wee1 | GGTGGGAGTTTAGCTGACG | TTGGGATTGAGGTTCGAGAT |
| Nlgn2 | GCAGAACCAGAGCGAGGAC | CGCCGTGTAGAAACAGCAT |
| Cav1 | GACGAGGTGAATGAGAAGCA | CACAGTGAAGGTGGTGAAGC |
| Agrn | GGCGGACCTACATCGAATACCT | GCCATGTAATCTGCACGTTCTG |
| Trh | CCTTGGATTCGGGAGTATTGC | TCCTGGAGTCTGCGAAGTGG |
| Nts | GATGTGAGAGTCCTGGAGGC | ATCCAGGACAAGGGGAAGTT |
| RT1-Da | TTGACTTTGACGGTGACGAG | CATTGGCATCTGGAGTGTTG |
